# Cluster analysis of replicated alternative polyadenylation data using canonical correlation analysis

**DOI:** 10.1186/s12864-019-5433-7

**Published:** 2019-01-22

**Authors:** Wenbin Ye, Yuqi Long, Guoli Ji, Yaru Su, Pengchao Ye, Hongjuan Fu, Xiaohui Wu

**Affiliations:** 1 Department of Automation, Xiamen University, Xiamen, 361005 China; 2 Software Quality Testing Engineering Research Center, China Electronic Product Reliability and Environmental Testing Research Institute, Guangzhou, 510610 China; 3 College of Mathematics and Computer Science, Fuzhou University, Fuzhou, 350116 China; 4 Innovation Center for Cell Biology, Xiamen University, Xiamen, 361005 China

**Keywords:** Alternative polyadenylation, Cluster analysis, Gene expression, Canonical correlation analysis, Network inference

## Abstract

**Background:**

Alternative polyadenylation (APA) has emerged as a pervasive mechanism that contributes to the transcriptome complexity and dynamics of gene regulation. The current tsunami of whole genome poly(A) site data from various conditions generated by 3′ end sequencing provides a valuable data source for the study of APA-related gene expression. Cluster analysis is a powerful technique for investigating the association structure among genes, however, conventional gene clustering methods are not suitable for APA-related data as they fail to consider the information of poly(A) sites (e.g., location, abundance, number, etc.) within each gene or measure the association among poly(A) sites between two genes.

**Results:**

Here we proposed a computational framework, named PASCCA, for clustering genes from replicated or unreplicated poly(A) site data using canonical correlation analysis (CCA). PASCCA incorporates multiple layers of gene expression data from both the poly(A) site level and gene level and takes into account the number of replicates and the variability within each experimental group. Moreover, PASCCA characterizes poly(A) sites in various ways including the abundance and relative usage, which can exploit the advantages of 3′ end deep sequencing in quantifying APA sites. Using both real and synthetic poly(A) site data sets, the cluster analysis demonstrates that PASCCA outperforms other widely-used distance measures under five performance metrics including connectivity, the Dunn index, average distance, average distance between means, and the biological homogeneity index. We also used PASCCA to infer APA-specific gene modules from recently published poly(A) site data of rice and discovered some distinct functional gene modules. We have made PASCCA an easy-to-use R package for APA-related gene expression analyses, including the characterization of poly(A) sites, quantification of association between genes, and clustering of genes.

**Conclusions:**

By providing a better treatment of the noise inherent in repeated measurements and taking into account multiple layers of poly(A) site data, PASCCA could be a general tool for clustering and analyzing APA-specific gene expression data. PASCCA could be used to elucidate the dynamic interplay of genes and their APA sites among various biological conditions from emerging 3′ end sequencing data to address the complex biological phenomenon.

**Electronic supplementary material:**

The online version of this article (10.1186/s12864-019-5433-7) contains supplementary material, which is available to authorized users.

## Background

Messenger RNA (mRNA) polyadenylation is an essential cellular process in eukaryotes, which consists of cleavage at the 3′ end of pre-mRNA and an addition of a tract of adenosines [poly(A) tail]. As one of the key post-transcriptional events, polyadenylation plays important roles in many aspects of mRNA biogenesis and functions, such as mRNA stability, localization, and translation [1, 2]. Accumulating genomic studies have indicated that most eukaryotic genes (more than 70% of genes in plants or mammals) can undergo alternative polyadenylation (APA) [3–7], leading to mRNAs with variable 3′ ends and/or different coding potentials [8, 9]. APA is now emerging as a pervasive mechanism that contributes to dynamics of gene regulation and links to important cellular fates. For example, APA can be regulated in a tissue- and/or developmental stage- specific manner. Global 3’ UTR shortening was observed in testis, proliferating cells, and cancer cells [3, 10, 11]. APA is also associated with flowering time in plants [12] and oncogene activation in human cancer cells [11]. Recent whole genome poly(A) site data from various conditions generated by 3′ end sequencing [7, 13–16] have stimulated interests in elucidating the dynamics of APA and its implications for regulation of gene expression, which can be a potential data source for the study of APA-related gene expression. Surprisingly, however, as data continue to accumulate, there is no general method or tool to analyze gene expression regarding APA regulation in different tissue types, developmental stages, or disease states.

Clustering is one of the most frequently used analyses on genomic data, which has been demonstrated to be a powerful technique for investigating the association structure among genes as well as underlying molecular mechanisms of gene clusters [17, 18]. The conventional cluster analysis is to apply widely used clustering algorithms on gene expression data, such as correlation or Euclidean distance based hierarchical clustering, K-means clustering, and Self Organizing Map [17, 19, 20]. However, traditional methods for clustering gene expression data are not suitable for APA-related gene expression analysis. First, in conventional gene cluster analyses, a single value, such as the raw count or FPKM (fragments per kilobase per million mapped fragments) [21], is used to represent gene expression level, while this is not applicable for the case of poly(A) site data as one gene can have multiple poly(A) sites. A common approach for analyzing gene expression from poly(A) site data is summing up the abundance of poly(A) sites within each gene and then applying popular clustering algorithms [22–24]. Although this is a simple and direct way, it would overlook the information of poly(A) sites (e.g., location, abundance, number, etc.) within each gene. Consequently, for example, the difference between two genes with different number of poly(A) sites but the same overall abundance was not considered in previous studies. As such, it is necessary to take into account the number, abundance, even the location of all poly(A) sites within each gene. Second, the result of a cluster analysis heavily depends on the cluster algorithm, especially the similarity measure between genes [17]. Distance measures such as correlation coefficients, Minkowski distance, and mutual information [17] have been widely employed in traditional cluster analyses, while such metrics are not able to measure the association among poly(A) sites between two genes. It is important but still challenging to design a measure to involve multiple layers of gene expression data from both the poly(A) site level and gene level. Third, although the regulation of APA across different physiological or pathological conditions has been well studied in recent years [7–9, 25, 26], cluster analysis using poly(A) site data has not been extensively studied in the field of APA. Most previous studies on APA focused on the analyses of 3’ UTR lengthening or shortening across various tissues or development stages [7, 23, 26–28], while the analysis of gene expression is scarce. Recent advances in deep 3′ end sequencing have provided multiple layers of transcriptome complexity detailing individual poly(A) sites within each gene rather than just overall gene expression [6, 7, 15, 24, 25, 29], placing new demands on the methods applied to identify potential gene modules associated with specific APA regulation.

The reliability of the biological conclusion drawn from genomic studies heavily depends on the quality of the biological data used, while in most cases, biological experiments are often subject to various potential sources of variance. To reduce the inherent noise as well as produce reproducible and statistically significant results, a common approach is to conduct repeated measurements (replicates). Replication is important for statistics analysis as it can not only enhance the precision of estimated quantities but also provide information about the random fluctuation or the uncertainty of the derived estimate [30]. As the cost of deep sequencing is declining, growing genomic data are being generated with repeated measurements. Conventional clustering algorithms such as k-means or hierarchical clustering are not ideal to deal with repeated data as they ignore the specific experimental design under which the biological data were collected. In most gene expression analyses, gene expression levels of different replicates are first averaged and then analyzed with conventional clustering algorithms, which fails to employ the information concerning the variability among replicates. Considering variability in gene expression analysis would help to increase the detection power [31] and yield clusters with higher accuracy and stability [32]. With this in mind, several clustering methods or distance measures have been proposed for summarizing repeated measurements, such as confidence interval inferential methodology [30], the multivariate correlation coefficient method [33, 34], and infinite mixture model-based approach [32]. However, these methods are not applicable for the APA-related gene expression data because each individual gene contains multi-layer information about poly(A) site usage and it cannot be treated as an independent feature. Recently, several methods or tools, such as RseqNet [35] and SpliceNet [36], were proposed to infer co-expression network from multi-layer genomic data taking into account the expression difference among exons and isoforms. However, these methods fail to take into consideration the variance among multiple replicates and are not specialized for APA analyses. Whole genome poly(A) site data with replicates across various tissues and/or developmental states are being generated [7, 13, 14], demanding computationally efficient methods to take advantage of these new data sets. Incorporating both repeated measurements and APA knowledge into the analysis of gene expression regulation would lead to more statistically significant and biologically relevant insights in the field of APA.

Here we proposed a computational framework, named PASCCA, for clustering genes from poly(A) site data using canonical correlation analysis (CCA). PASCCA is intended to leverage the merit of existing poly(A) site data for APA-related gene expression analyses, which has the following advantages. First, PASCCA incorporates detailed information about APA sites within each gene, which can quantify the overall association of APA sites across various conditions between each pair of genes. Second, PASCCA takes into account both the number of replicates and the variability within each experimental group, which is capable of fully exploring the similarity between repeated measures. Third, PASCCA characterizes poly(A) sites in various ways including the abundance and relative usage, which can exploit the advantages of 3′ end deep sequencing in quantifying APA sites. Moreover, PASCCA provides a correlation measure rather than a clustering method, which could be easily used as a similarity metric for various clustering methods, gene network inference methods, or other potential circumstances. We have made PASCCA an easy-to-use R package for analyses of APA-related gene expression. Using both real and synthetic poly(A) site data sets, the cluster analysis demonstrates that PASCCA performs better than other widely-used distance measures under several performance metrics including connectivity, the Dunn index, average distance, average distance between means, and the biological homogeneity index. We also used PASCCA to infer APA-specific gene modules from a recently published poly(A) site data set of rice [7] and discovered some distinct functional gene modules. By providing a better treatment of the noise inherent in repeated measurements and taking into account multiple layers of poly(A) site data, PASCCA could be a general tool for clustering and analyzing APA-specific gene expression data.

## Results

### Overview of PASCCA

**Fig. 1:**
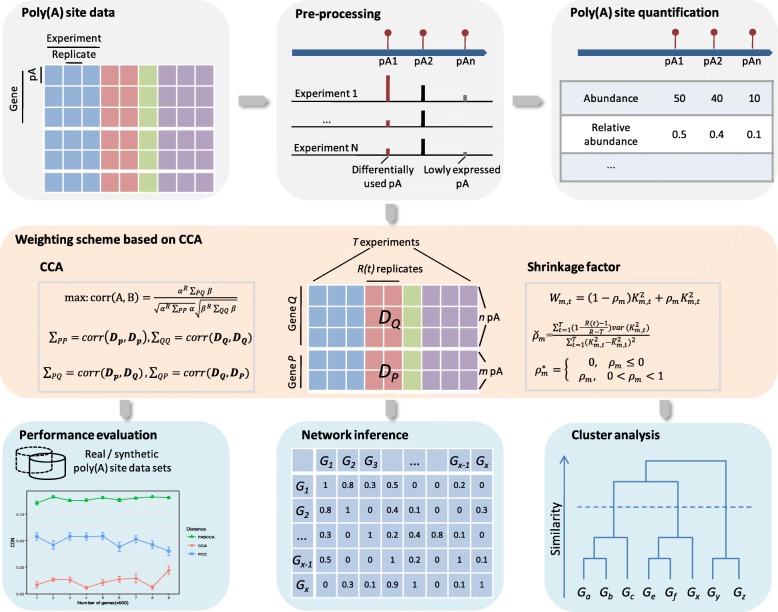
General pipeline of PASCCA

PASCCA consists of a general pipeline for analyzing poly(A) site data (Fig. 1). First, poly(A) site data are pre-processed for further APA-specific gene expression analyses. Poly(A) sites with low abundance, sites located in intergenic regions, or genes that possess single poly(A) site are removed. The retained poly(A) sites are subjected to DEXseq [37] to identify poly(A) sites with differential usage among experiments and sites that are not differentially used in at least one pair of experiments are discarded. Next, different quantification methods can be used to characterize each poly(A) site. In addition to using the abundance to represent each poly(A) site, we included the relative usage as another metric to quantify poly(A) sites, which has been reported critical in the determination of poly(A) site choice among different conditions [5]. After quantifying poly(A) sites, the data are then subjected to a weighting scheme based on canonical correlation analysis to obtain the correlation between each gene pair. As the core step of PASCCA, this weighting scheme incorporates detailed information about poly(A) sites within each gene and takes into account both the number of replicates and the variability within each experiment. The output of this step is a similarity matrix which can be used for downstream analyses, such as clustering and network inference. Both real and synthetic poly(A) site data sets were tested and various performance indexes were employed for comprehensive performance evaluation of PASCCA.

### Evaluation of PASCCA on real poly(A) site data set in rice

**Fig. 2:**
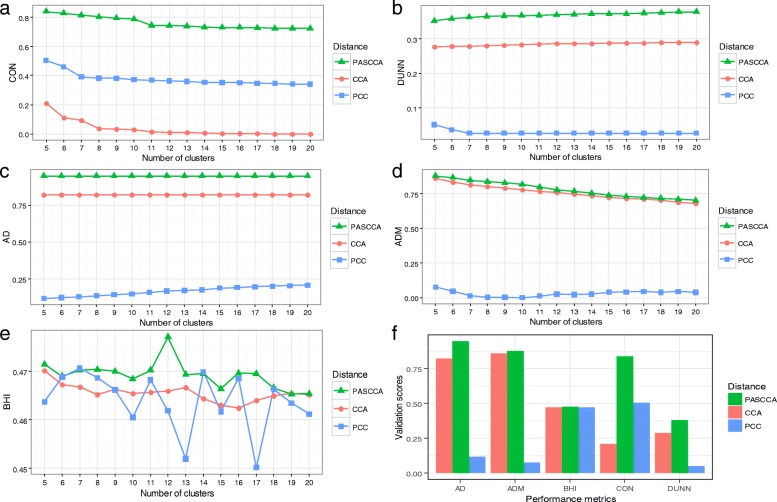
Evaluation of PASCCA on real poly(A) site data in rice using hierarchical clustering. Standardized cluster validation scores for various performance indexes with increasing number of clusters were calculated, including CON (**a**), DUNN (**b**), AD (**c**), ADM (**d**), and BHI (**e**). Without knowing the true number of clusters in a given data set, variable number of clusters ranging from 5 to 20 was set. Comparison of performances with the estimated number of clusters for each method was shown in (**f**). Larger score indicates better performance. CON, connectivity; DUNN, Dunn index; AD, average distance; ADM, average distance between means; BHI, biological homogeneity index

We adopted a replicated poly(A) site data set from rice to evaluate PASCCA, which consists of 14 tissues each with two or three repeated measurements [7]. First we identified 4564 genes with at least one differentially used poly(A) site using DEXseq [37], and 14,107 poly(A) sites in these genes were obtained for further analysis. The weight matrix obtained from PASCCA was used as the distance matrix and compared with other correlation-based distance metrics, including Pearson’s correlation coefficient (PCC) and CCA. Since no priori knowledge of the exact number of clusters was available for the real rice poly(A) site data, variable number of clusters ranging from 5 to 20 was set for performance evaluation. Under each specific number of clusters, the performance of each distance measure was assessed by calculating various performance metrics based on the hierarchical clustering method. PASCCA shows the best performance among all distance measures regardless of performance metrics employed (Fig. 2). The performance of PASCCA is consistently higher than PCC and CCA in terms of the internal validation measures, CON (connectivity) and DUNN (the Dunn index) (Fig. 2a and b), indicating that the variance within clusters derived from PASCCA is much smaller than that from PCC and CCA. Considering the stability validation, PASCCA is apparently superior to PCC and has slight advantages over CCA (Fig. 2c and d). PASCCA also provides the most biologically relevant clustering partitions as measured by the biological homogeneity index (BHI) (Fig. 2e), reflecting the increased biological homogeneity of clusters obtained from PASCCA. Generally, PCC provides the worst results, which may be due to that PCC fails to incorporate detailed information of poly(A) sites within each gene. Next, instead of choosing variable number of clusters, the best number of clusters for each distance measure was estimated by the Silhouette criterion [17, 38]. Still, PASCCA shows overall better performance than PCC and CCA (Fig. 2f), demonstrating that clusters identified from PASCCA are more physically stable and compact.

### Evaluation of PASCCA on synthetic poly(A) site data sets

**Fig. 3:**
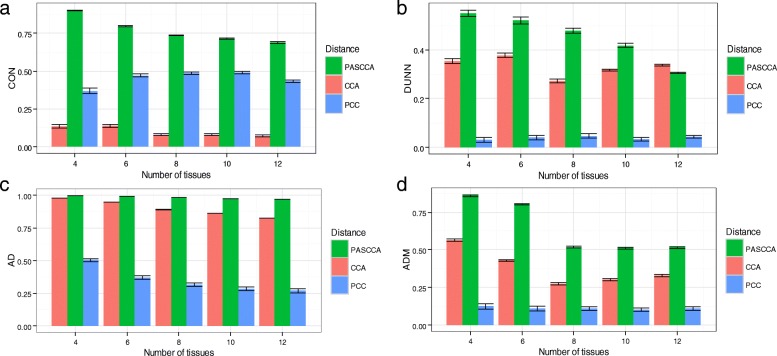
Validation scores on synthetic data sets with different number of experiments using hierarchical clustering. Standardized cluster validation scores for various cluster validation measures across a range of different number of clusters were calculated, including CON (**a**), DUNN (**b**), AD (**c**), and ADM (**d**). For each trial with a fixed number of experiments, ten data sets were randomly selected from the whole synthetic data set. The best number of clusters was estimated for each trial. The mean validation scores for trials performed on the 10 random data sets were plotted. The standard deviation is depicted as an error bar

To further demonstrate the superiority of PASCCA on repeated data, we analyzed synthetic data sets with replicates (see Methods). We applied PASCCA to three different kinds of data sets with variable number of experiments, genes, and repeated measurements. We need to point out that, there is no real gene in the synthetic data sets, therefore the index of BHI was not considered in the simulation study. In the first simulation study, we tested synthetic data sets with different number of experiments. Given a specific number of experiments ranging from four to twelve, ten synthetic data sets each with 500 genes that possess multiple poly(A) sites and three replicates for each experiment were generated. For each run of clustering, we set the number of clusters varying from 5 to 20. After clustering ten synthetic data sets of a given number of experiments, we obtained a total of 160 validation scores for each performance metric under one distance. Then the mean and standard deviation of the 160 validation scores were calculated. In almost all cases, PASCCA presents the best results, followed by CCA (Fig. 3). Considering the internal metrics (CON and DUNN), PASCCA outperforms CCA and PCC (Fig. 3a and b), reflecting higher compactness, connectedness, and separation of cluster partitions obtained from PASCCA. Particularly, PCC provides better performance than CCA regarding the CON metric (Fig. 3a) whereas CCA outperforms PCC regarding the DUNN metric (Fig. 3b), which reflects that PCC generates cluster partitions with higher connectedness while CCA generates cluster partitions with higher separation. When considering the AD (average distance) metric, PASCCA has a slight advantage over CCA but provides far better performance than PCC (Fig. 3c), reflecting the smaller average distance between observations in the same cluster obtained from PASCCA or CCA than that from PCC. Regarding the ADM (average distance between means) metric, again, PASCCA has the best performance, followed by CCA, and PCC provides the worst results (Fig. 3d).

In the second simulation study, we tested synthetic data sets with variable number of genes to assess the effect of data size on clustering. Given a restricted number of genes ranging from 500 to 4500 with an increment of 500, ten data sets each with 14 experiments and three replicates for each experiment were randomly generated. Similar to the scenario on different number of experiments, we obtained the mean and standard deviation for each performance metric under each distance measure. Again, PASCCA provides the best results regardless of performance metrics or number of genes (Additional file 1: Figure S1). The variance within clusters obtained from PASCCA is much smaller than that from PCC and CCA, which is reflected by metrics of CON and DUNN (Additional file 1: Figures S1a and b). According to metrics of AD and ADM, PASCCA also provides more stable results than PCC and CCA (Additional file 1: Figure S1c and d).

In the third evaluation scenario, we generated synthetic data sets that contain 500 genes and 14 experiments with two to 15 replicates for each experiment. Regarding CON and AD metrics, PASCCA presents consistently higher performance than CCA and PCC, whereas CCA and PCC provides the worst results according to CON and AD, respectively (Additional file 1: Figure S2a and c). Interestingly, regarding the AD metric, the performance of CCA is decreased with the increase of the number of replicates while the performance of PASCCA is high and stable (Additional file 1: Figure S2c), demonstrating the importance of considering replicates in clustering. Considering the DUNN and ADM metrics, PASCCA performs slightly worse or equally to CCA when the number of replicates is low, while PASCCA outperforms CCA with the increase of the number of replicates (Additional file 1: Figure S2b and d). Overall, PASCCA stands out as the best distance, while PCC provides the worst performance.

### Characterization of poly(A) sites by relative abundance

**Fig. 4:**
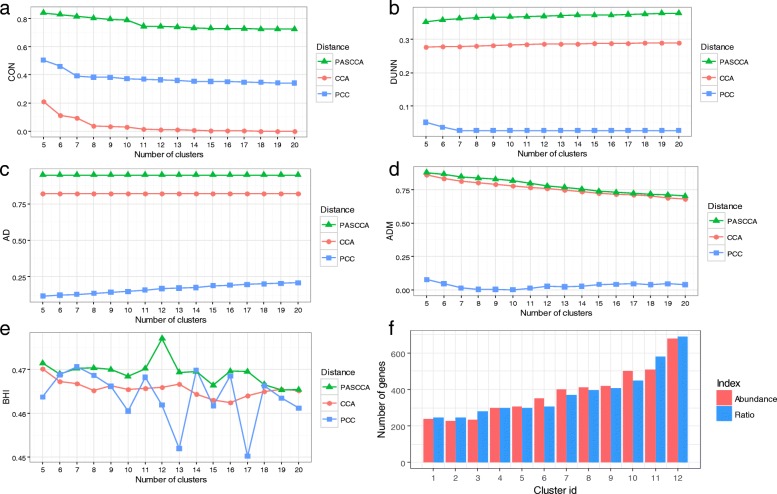
Cluster analyses of real poly(A) site data set based on relative abundance. Standardized cluster validation scores for various performance indexes with the increasing of the number of clusters were calculated, including CON (**a**), DUNN(**b**), AD (**c**), ADM (**d**), and BHI (**e**). Larger scores indicate better performance. Without knowing the true number of clusters in a given data set, variable number of clusters ranging from 5 to 20 was set. (f) Number of genes in clusters obtained from PASCCA using poly(A) site data set characterized by abundance or relative abundance

A previous study [5] used the relative proportion of reads rather than the number of reads of poly(A) sites to determine the poly(A) site choice between two conditions and found a large number of Arabidopsis genes were altered in the *oxt6* mutant. Here we used the relative abundance of the poly(A) site as another metric to characterize poly(A) sites. Given a gene with *n* poly(A) sites in one experiment, the relative abundance for poly(A) site *p* is $\frac{a(p)}{\sum_na(i)},i=1..n$, where *a*(*p*) is the abundance of poly(A) site *p*. Using the real poly(A) site data set represented by the relative abundance, we obtained weights for all gene pairs using PASCCA. First, we conducted the cluster analysis to evaluate the performance of PASCCA. Again, PASCCA is superior to CCA and PCC regardless of performance metrics (Fig. 4a-e). Considering the internal validation metrics, PASCCA apparently outperforms CCA and PCC (Fig. 4a and b), which is similar to the result using the abundance of poly(A) sites (Fig. 2a and b). Regarding the stability validation metrics, PASCCA has slight advantages over CCA using the AD metric whereas they have comparable performance according to the ADM metric (Fig. 4c and d). Still, both PASCCA and CCA clearly outperform PCC. In terms of the BHI metric, PASCCA presents the best results, followed by PCC, while CCA provides the worst results (Fig. 4e). Obviously, regardless of ways to characterize poly(A) sites, PASCCA generally outperforms PCC and CCA (Figs.2 and 4). According to the BHI metric, both ways present the best performance when the number of clusters is 12 (Figs. 2e and 4e). In the case with 12 clusters, distributions of numbers of genes in each cluster obtained from both ways are similar (Fig. 4f). Surprisingly, however, less than 30% of genes in clusters from both ways are overlapped (Additional file 1: Figure S3). For example, for the largest cluster that has ~ 700 genes from both ways, only 195 genes are overlapped. These results suggest that different ways used to characterize poly(A) sites may contribute considerably to the clustering results, therefore, it is critical to choose the way for representing poly(A) sites and to carefully inspect the clustering results according to the respective biological questions.

### Distinct gene modules identified by network inference integrating PASCCA

Network inference has become a critical step towards understanding complex biological phenomena. Next, we demonstrated the use of PASCCA in constructing APA-specific gene networks. First weights for all gene pairs were obtained from PASCCA and CCA, respectively. Only gene pairs with statistically significant weights were retained. The weight matrices from both methods were further used as adjacency matrices for WGCNA [39], a popular R package for weighted correlation network analysis, to infer network modules. For comparison, we also obtained network modules based on gene expression levels that were obtained by summing up reads of all poly(A) sites in each gene (hereinafter referred to as genePCC). Each module obtained from WGCNA can be considered as a co-expression network. Using WGCNA, nine, eight, and 15 modules were obtained using PASCCA, CCA, and genePCC, respectively (Additional file 1: Figure S4a). Although PASCCA and CCA obtained similar number of modules, the number of genes in these modules varied widely. Particularly, among the eight modules obtained from CCA, the vast majority of genes (61%, 2768) were found in one module. In contrast, genes are more evenly distributed in modules obtained from PASCCA (Additional file 1: Figure S4a). It is possible that CCA failed to distinguish small modules from large ones and consequently produces an overbalanced module with large number of genes. We also found that ~ 60% of genes from each module obtained from PASCCA are overlapped with the largest module obtained from CCA (Additional file 1: Figure S4b), indicating that PASCCA is capable of segmenting a large group of genes by incorporating information such as the variance among replicates. Among the three methods, the highest number of modules (15) were obtained by genePCC. Similar to CCA, the numbers of genes in modules from genePCC are also very unevenly distributed, ranging from 65 to 1261.

**Fig. 5:**
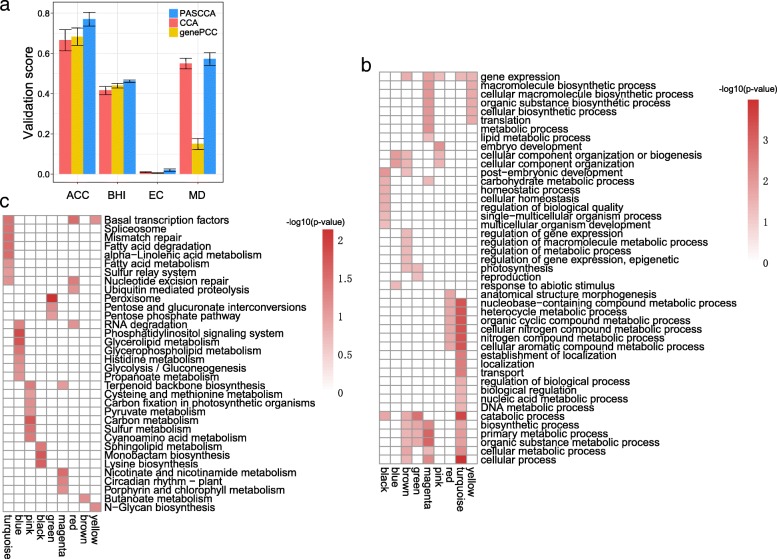
Analyses of gene modules. **a** Validation scores of network metrics for gene modules identified by genePCC, CCA, and PASCCA. Larger scores indicate better performance. Bars correspond to the mean validation scores for all modules identified by the corresponding method. The standard deviation is depicted as an error bar. **b** Gene ontology result for modules obtained from PASCCA. **c** KEGG pathway enrichment analysis for modules obtained from PASCCA

In order to evaluate the performance of PASCCA in the network construction, we also calculated various metrics for assessing the modularity and community structure in a network. Generally, PASCCA has better performance than CCA and genePCC regardless of network metrics employed (Fig. 5a). The increased density of modules obtained from PASCCA is reflected in a higher ACC (average clustering coefficient) score of 0.77, compared to 0.67 in CCA and 0.68 in genePCC. According to the BHI metric, modules generated from PASCCA are more biologically meaningful than those from CCA or genePCC. Particularly, genePCC has much lower score of MD (module degree) metric (0.15) than PASCCA (0.57) or CCA (0.55), reflecting that there are much denser connections between nodes within modules but much sparser connections between nodes in different modules obtained from PASCCA or CCA than from genePCC.

Next, we examined the relationship between tissues and modules identified by PASCCA according to the correlation between each pair of module and tissue. These modules can be largely divided into two groups (Additional file 1: Figure S5): one group is of high correlation with tissues of dry seed, endosperm, imbibed seed, and embryo; the other group is highly correlated with tissues of shoot, leaf, stem, and mature pollen. This result reflects that different developmental stages of the same tissues have similar distribution of module correlations, which is consistent with the previous result from rice that different developmental stages of the same tissues have similar expression patterns of transcript isoforms [7]. We also performed GO (gene ontology) analysis and KEGG (Kyoto encyclopedia of genes and genomes) pathway enrichment analysis for genes or hub genes from these modules. GO analysis revealed distinct functions associated with different modules (Fig. 5b). For example, the pink module is uniquely enriched in the biological process of embryo development; the red module is exclusively enriched in the biological process of anatomical structure morphogenesis. Green, brown and magenta modules are over-represented in processes such as carbohydrate metabolic process, biosynthetic process, photosynthesis, and lipid metabolic process, which play critical roles in controlling plant growth, development, and crop yield [40, 41]. We also performed KEGG pathway enrichment analysis for hub genes identified from each individual modules to investigate their functional importance. Hub genes for a module were defined as genes with correlation values with the respective module larger than 0.7. Hub genes for all modules obtained from PASCCA were provided in Additional file 2. Turquoise and pink modules are over-represented in pathways of cysteine and methionine metabolism and sulfur relay system, which were associated with pathways of glutathione (GSH) metabolism and biosynthesis of its precursor (Fig. 5c). GSH has been found to be critical for plant cold acclimation and chilling tolerance through reducing the accumulation of reactive oxygen species [42, 43]. It is possible that the APA-mediated GSH metabolism may be crucial in the adaptation of rice to extreme temperate climates. The blue module is exclusively enriched in the pathway of phosphatidylinositol signaling system, which is the main signaling pathway of plant disease resistance. The green, pink and magenta modules are over-represented in plant growth-related pathways, including pentose phosphate pathway, porphyrin and chlorophyll metabolism, carbon metabolism, carbon fixation in photosynthetic organisms and circadian rhythm - plant. These findings indicated that these APA-mediated gene modules may play an important role in the growth process of rice.

## Discussion

Cluster analysis has been enormously successful in the past decades in detecting patterns or relationships between genes to reveal the underlying molecular mechanism [17, 19, 20, 44]. Inference of isoform or protein networks has also attracted considerable attention recently, and several tools now exist for network construction involving single or multiple layers of biological information [35, 36, 45–48]. Despite of the availability of various clustering and network inference methods, integrating appropriate distance measures for different biological data sets or research purposes is one of the primary issues [44]. Provided that most clustering methods use a distance matrix as the input, choosing a distance measure employed by the clustering method is a non-trivial task which can significantly affect the final clustering performance [17]. Recent genomic studies have uncovered widespread occurrences of APA and found a large number of genes with APA sites [8, 24, 49, 50], however, methods of cluster analysis or network inference for APA-related gene expression data are still scarce, placing demands on developing new methods complementary to traditional APA analyses to gain deeper insights into the underlying biological system. Here, we resorted to the method of canonical correlation analysis and assigned the weight for each gene pair that represents the strength of direct interaction between genes. Especially, our model enhances weights of direct interactions by incorporating various poly(A) site information and repeated measurements of biological experiments.

In this study we have proposed PASCCA, a new pipeline for clustering and network inference from APA-related gene expression data with/without repeated measurements. The weight matrix from the weighting scheme can be an alternative to the similarity or distance metric for downstream cluster analysis, network inference, and other possible purposes. CCA, a traditional statistical method for investigating the relationships between two sets of variables, have recently been employed in genomic studies to estimate the correlations from gene expression data, however, the use of CCA is still fairly limited [35, 51]. As one kind of correlation coefficient, CCA is useful in cluster analysis to determine the correlation between genes. However, when CCA was applied for clustering gene expression, replicates of each treatment group were simply averaged regardless the underlying variance. This is, unfortunately, not fully applicable on the current situation that genomic data with multiple biological replicates are increasingly generated to produce reproducible and statistically significant results. To meet these specific needs, PASCCA seamlessly integrated the concept of shrinkage and CCA to improve the estimation of correlation for data with replicates. Shrinkage concept has been widely employed in previous studies to overcome limitations of Pearson’s correlation coefficient [33, 52–54]. By incorporating shrinkage concept, PASCCA is capable of taking into account both the number of repeated measurements and the variance within each experiment, which can better cluster replicated biological data and highlight new information pertaining to gene expression patterns. Using one of the most popular cluster methods, hierarchical clustering, we comprehensively compared PASCCA with two correlation based distance measures including CCA and PCC, based on diverse cluster and network validation metrics. Results demonstrated that PASCCA can generate clusters and networks with higher quality using both synthetic and real poly(A) site data sets. Moreover, we test different synthetic data sets with variable number of experiments, genes, and repeated measurements, results showed that PASCCA has higher performance and better robustness than other distances investigated. One of the major reasons for the superiority of PASCCA is that the shrinkage factor introduced in the CCA benefits an optimal estimate of the error in replicates and thus can better quantify the relationship between genes, even for data with small number of replicates which is the normal case in many genomic studies.

Another compelling feature of PASCCA is, perhaps, incorporating detailed information about APA sites to quantify the association between genes. Previously, to measure the gene expression level from 3′ end sequencing, overall gene expressions were determined by summing up all poly(A) sites within each gene [22–24]. Although the recent high-throughput 3′ end sequencing has made detailing APA sites cost-effective, the true merit of 3′ sequencing in quantifying APA sites has not been fully explored in most poly(A) studies to date. Unlike traditional distances such as the correlation coefficient and Euclidean distance that are based on overall gene expressions without considering biological details within each gene, PASCCA is capable of inferring the multivariable (APA sites) dependency between two genes. By comparing the clustering results from PASCCA with other two correlations including PCC and CCA, we demonstrated that PASCCA can significantly improve the clustering performance by quantifying abundance difference in APA sites. More importantly, PASCCA provides an advantage for full exploitation of poly(A) sites by incorporating different metrics in quantifying APA sites before conducting the cluster analysis or network inference. Consequently, the performance of PASCCA may be affected by the quantification metric used. To assess the influence of different quantification metrics, we conducted two cluster analyses on the same real poly(A) site data set but using two different metrics, abundance and relative abundance. Experimental results using different quantification schemes vary greatly. Numerous studies have emphasized the importance of relative usage instead of the abundance of poly(A) site in determining poly(A) site choice among different conditions [5, 24]. For practical application purpose, we suggest using both quantification metrics for weighting gene pairs which could be complimentary to each other.

Given the importance of APA in regulating gene expression, the lack of methodology for quantifying the correlation in gene pairs is one of the big hurdles in the construction of APA-specific biological networks. PASCCA also contributes considerably to providing a correlation measure rather than a clustering method, which could be easily used as a similarity metric for downstream cluster analyses or network inference. Using the latest real poly(A) site data set, we adopted WGCNA [39] to infer APA-specific gene expression networks based on the weight matrix calculated from PASCCA in order to demonstrate the biological importance of PASCCA and its implications on APA studies. We discovered nine distinct gene modules across 14 different tissues and developmental stages of rice. GO analysis suggests that some gene modules are strongly involved in biological processes relevant to plant growth processes including lipid metabolic process, photosynthesis, biosynthetic process, and carbohydrate metabolic process (Fig. 5b). Similarly, KEGG enrichment analysis showed that these modules were significantly enriched in plant growth-related pathways, such as the pentose phosphate pathway, porphyrin and chlorophyll metabolism, carbon fixation in photosynthetic organisms (Fig. 5c). These findings indicated that gene modules inferred from PASCCA may play an important role in the growth process of rice. In addition, we found some gene modules were over-represented in pathways of GSH metabolism and biosynthesis of its precursor (Fig. 5c) which may be crucial for plant chilling tolerance and cold acclimation [42, 43], suggesting that genes involved in these pathways may be functionally important in the adaptation of rice to extreme temperate climates. These results showed the potential of incorporating PASCCA to yield important gene modules and to lead to testable hypotheses in biology.

## Conclusions

We proposed a computational framework, called PASCCA, for analyses of APA-related gene expression, including the characterization of poly(A) sites, quantification of association between genes with/without repeated measurements, clustering of APA-related genes to infer significant APA specific gene modules, and the evaluation of clustering performance with a variety of indexes. PASCCA incorporates multiple layers of gene expression data from both the poly(A) site level and gene level and takes into account both the number of replicates and the variability within each experimental group. PASCCA could be a general tool for clustering and analyzing APA-specific gene expression data, which is useful in elucidating the dynamic interplay of genes and their APA sites among various biological conditions from emerging 3′ end sequencing data to address the complex biological phenomenon.

## Methods

### Real and synthetic poly(A) site data sets

We used both real and synthetic data sets to evaluate PASCCA. The real poly(A) site data set which consists of 14 tissues each with two or three repeated measurements in rice was collected from the previous study [7]. Fu et al. focused on the identification of tissue specific poly(A) sites among different tissues but did not conduct any cluster analysis to infer APA-specific gene modules. This data set contains a total of 68,220 poly(A) sites dispersed in 28,032 genes, which is the largest poly(A) site data set in plants to date. To obtain poly(A) sites with high confidence, we discarded poly(A) sites that are supported by less than five reads.

It is noteworthy that data sets with a certain number of conditions (tissues, developmental states, etc.) and repeated measurements are required for fully evaluating PASCCA, unfortunately, very few publicly available poly(A) site data sets meet both criteria. Although there are plenty of gene expression data with repeated measurements from microarray or RNA-seq, repeated poly(A) site data sets are still rare. To overcome these limitations, we used a two-step process to generate synthetic data that have the same distribution of abundance of poly(A) sites derived from the rice data. Given a gene *g* with *k* poly(A) sites, let the abundance of poly(A) site *i* (*i* = 1. . *k*) be *a*(*i*), then the true frequency of poly(A) site *i* is *p*(*i*) = *a*(*i*)/ ∑ *a*(*k*). For each experiment, we generated the simulated abundance of each poly(A) site in each gene according to the binomial distribution with probability being *p*(*i*) and size being ∑*a*(*k*). To simulate replicates for each experiment, we added random noises based on the normal distribution using the R function *rnorm* with both the mean and standard deviation derived from the true data set. To evaluate the performance of PASCCA, data sets with different number of experiments (tissues), repeated measurements, and genes were randomly selected from the synthetic data set for evaluation.

### Performance evaluation

For the performance evaluation, our primary interest lies in the comparison of the distance measure provided in PASCCA with other distance measures rather than on the assessment of clustering methods. Because that distance measures are normally employed with a clustering method but not as a single entity, we applied one of the most popular clustering methods, hierarchical clustering (HC) [55] and compared PASCCA with two distance measures, including PCC and CCA [35]. The reason for choosing PCC and CCA for comparison is that they are both the correlation based distances used in biological data which are the same kind of distance as PASCCA. PCC is one of the most popular distances in cluster analysis of gene expression data. Hong et al. [35] proposed a CCA-based method and developed a package called RSeqNet for clustering genes by taking into account the expression difference among exons. Although RSeqNet was not initially developed for poly(A) site data, it can be used for calculating the correlation between genes using the processed and formatted poly(A) site data. It should be noted that PCC is not capable of incorporating the poly(A) site information of genes, therefore, we summed up the abundance of all poly(A) sites within a gene as the expression level for that gene before applying PCC for clustering.

There is no priori knowledge of what genes should be clustered together according to the poly(A) site data. We then used several performance metrics that do not require the class label to quantitatively assess the overall performance of PASCCA, which cover three main types of cluster validation measures including internal, stability, and biological [56]. The internal validation evaluates the quality of the clustering based on intrinsic information in the data, using only the data set and the clustering partition as input. For internal validation, we used measures that reflect the compactness, connectedness, and separation of the cluster partitions, including the connectivity (CON) and the Dunn index (DUNN) [44, 56]. The CON metric measures the extent of observations that are placed in the same cluster as their nearest neighbours in the data space; the DUNN metric reflects non-linear combinations of the compactness and separation [56]. The stability validation measures the stability of the clustering by comparing the clustering result between the full data and the perturbed data. For stability validation, we used two indexes including the average distance (AD) and the average distance between means (ADM) [44, 56]. The AD metric measures the average distance between observations clustered in the same cluster using the full data and the data with a single column removed; the ADM metric computes the average distance between cluster centers [56]. Biological validation measures the quality of the clustering by investigating the biological significance of clusters. We used the BHI (biological homogeneity index) to measure how biologically homogeneous a gene clustering is [56]. We adopted the R package clValid [56] to calculate validation scores for these metrics. As different metrics have different ranges of value, validation scores were normalized between 0 and 1 for a more intuitive comparison. The larger score indicates better performance.

To assess network modules identified by WGCNA [39] using different distance metrics, we employed several additional network metrics, including the eigenvector centrality (EC), module degree (MD), and average clustering coefficient (ACC) [57–60]. EC measures the influence of a node in a network. MD is a measure of the quality of the network module partition. ACC measures the density of triangles in a network.

### Weighting scheme based on canonical correlation analysis

We designed a weighting scheme based on CCA to quantify the correlation between each gene pair. We embedded the shrinkage correlation coefficient [33] into the CCA framework to infer the correlation between two genes by incorporating detailed layers of all poly(A) sites. Assuming that we have each gene measured across *T* experiments with *R*(*t*) replicates for the *t*^th^ experiment, then $R=\sum \limits_{t=1}^TR(t)$, where *R* is the total number of replicates of all experiments. Given a gene *G* with *K* poly(A) sites, let ${\boldsymbol{D}}_{\boldsymbol{G}}=\left\{{D}_{iG}^{\left( jr(j)\right)},i=1,\dots, K;j=1,\dots, T;r(j)=1,\dots, R(j)\right\}$ denote the set of measurements of all poly(A) sites in this gene, where ${D}_{iG}^{\left( jr(j)\right)}$ is the measurement for the *i*^th^ poly(A) site of gene *G* at the *r*(*j*)^th^ replicate of the *j*^th^ experiment. Given two genes *P* and *Q* each with *m* and *n* poly(A) sites (assuming *m* ≤ *n*), the objective is to quantify their relationship based on ***D***_***P***_ and ***D***_***Q***_. We adopted CCA to obtain the maximum correlation coefficients for ***D***_***P***_ and ***D***_***Q***_ by seeking weights *α* and *β* for ***D***_***P***_ and ***D***_***Q***_ which results in the maximun correlation coefficient for the linear combination of the *m* poly(A) sites in gene *P*, *A* = *α*^*R*^***D***_***P***_ and the linear combination of the *n* poly(A) sites in gene *Q*, *B* = *β*^*R*^***D***_***Q***_**.** This is equivalent to solving the following problem:

1 $$ {\displaystyle \begin{array}{c}\max :\mathrm{corr}\left(\mathrm{A},\mathrm{B}\right)=\frac{\alpha^R{\sum}_{PQ}\beta }{\sqrt{\alpha^R{\sum}_{PP}\alpha}\sqrt{\beta^R{\sum}_{QQ}\beta }}\\ {}{\sum}_{PP}= corr\left({\boldsymbol{D}}_{\boldsymbol{p}},{\boldsymbol{D}}_{\boldsymbol{p}}\right),{\sum}_{QQ}= corr\left({\boldsymbol{D}}_{\boldsymbol{Q}},{\boldsymbol{D}}_{\boldsymbol{Q}}\right)\\ {}{\sum}_{PQ}= corr\left({\boldsymbol{D}}_{\boldsymbol{p}},{\boldsymbol{D}}_{\boldsymbol{Q}}\right),{\sum}_{QP}= corr\left({\boldsymbol{D}}_{\boldsymbol{Q}},{\boldsymbol{D}}_{\boldsymbol{P}}\right).\end{array}} $$

Here ∑are the correlation matrices of samples.To obtain ∑_*PP*_, ∑_*PQ*_, ∑_*QQ*_,  and∑_*QP*_,we solved the correlation coefficient matrix for the *m* poly(A) sites in gene *P* and *n* poly(A) sites in gene *Q*. PCC is one of the most popular ways to calculate the correlation between two vectors, however, using the average value of each experiment or considering each replicate as an independent experiment would neglect the information concerning the variance among replicates. Considering the between-replicate variance, Yeung et al. proposed the standard deviation weighted correlation coefficient (SDCC) to model the variability of replicates, which showed higher accuracy and stability than using PCC [32]. However, when the number of replicates is much smaller relative to the number of genes, SDCC could be inaccurate in estimating errors [33]. Due to high labour and time costs, microarray or RNA-seq experiments are usually measured with limited number of replicates (e.g., < 5), SDCC is unfortunately not suitable for general gene expression data. To avoid the inaccuracy introduced by the small number of replicates, here we employed the shrinkage correlation coefficient (SCC) which has been applied in the analysis of replicated microarray data [33]. SCC can fully exploit the similarity between replicates for the robust statistical estimation of errors of replicated expression data.

Given *T* experiments, the real squared measurement errors of these experiments are denoted as *δ*(1), *δ*(2), … , *δ*(*T*). Initially, a *T*-dimensional model is required to estimate these *T* parameters, however, estimating parameters based on higher dimension would produce higher variance on the same data set. To reduce the estimation error, we projected the original *T*-dimensional model to the restricted one-dimensional sub model by using the mean of these *T* parameters,$\Theta (t)=\frac{1}{T}\sum \limits_{t=1}^T\delta (t)$. However, another type of estimation error would be introduced if we simply replace *δ*(1), *δ*(2), … , *δ*(*T*) with Θ(*t*). To balance both types of errors, we adopted the shrinkage error estimate. Given ${D}_{mG}^{tr(t)}$ as the measurement for the *m*^th^ poly(A) site of gene *G* at the *r*(*t*)^th^ replicate of the *t*^th^ experiment, ${\overline{E}}_{m,t}=\sum \limits_{r(t)=1}^{R(t)}{\mathrm{D}}_{\mathrm{mG}}^{\mathrm{tr}\left(\mathrm{t}\right)}/R(t)$ is the average value of all replicates of this poly(A) site in the *t*^th^ experiment and ${K}_{m,t}^2=\frac{1}{R(t)-1}\sum \limits_{r(t)=1}^{R(t)}{\left({D}_{mG}^{tr(t)}-{\overline{E}}_{m,t}\right)}^2$ is the variance of the *m*^th^ poly(A) site in the *t*^th^ experiment.

If the standard deviation (SD) is used as an estimate of the measurement error, then the SD-weighted average expression of poly(A) site *m* over the experiment *t* is:

2 $$ {\overline{E}}_{m,t}^{SD}={\sum}_{t=1}^T\frac{{\overline{E}}_{m,t}}{K_{m,t}^2}/{\sum}_{t=1}^T\frac{1}{K_{m,t}^2}. $$

To overcome the limitation of the SDCC method [32], we introduced the shrinkage error to substitute the mean square error in eq. (2) for a more accurate estimation of errors. We defined the unbiased estimate of the squared measurement error as

3 $$ {\overline{K}}_{m,t}^2={\sum}_{t=1}^T\frac{R(t)-1}{R-T}{K}_{m,t}^2. $$

We then defined a balanced estimate based on the linear regularization model [33]:

4 $$ {W}_{m,t}=\left(1-{\rho}_m\right){K}_{m,t}^2+{\rho}_m{\overline{K}}_{m,t}^2, $$

where *ρ*_*m*_*ϵ*[0, 1] is the shrinkage factor.

Next, the shrinkage factor can be estimated by the quadratic loss function [61, 62]:

5 $$ {\overset{\check{} }{\rho}}_m=\frac{\sum_{t=1}^T\left(1-\frac{R(t)-1}{R-T}\right)\mathit{\operatorname{var}}\left({K}_{m,t}^2\right)}{\sum_{t=1}^T{\left({K}_{m,t}^2-{\overline{K}}_{m,\mathrm{t}}^2\right)}^2}, $$

where $$ \operatorname{var}\left({K}_{m.t}^2\right)=\frac{R(t)}{{\left(R(t)-1\right)}^3}\sum \limits_{r(t)=1}^{R(t)}{\left[{\left({D}_{mG}^{tr(t)}-{\overline{E}}_{m,t}\right)}^2-{K}_{m,t}^2\right]}^2 $$.

To restrict *ρ*_*m*_ between 0 and 1, the final shrinkage factor is

6 $$ {\rho}_m^{\ast }=\left\{\begin{array}{c}0,\kern0.5em {\rho}_m\le 0\\ {}\ {\rho}_m,\kern0.5em 0<{\rho}_m<1\\ {}\begin{array}{cc}1,& {\rho}_m\ge 1\end{array}\end{array}\right.. $$

Substituting ${\rho}_m^{\ast }$ into eq. (4), the balanced estimate is

7 $$ {W}_{m,t}^{\ast }=\left(1-{\rho}_m^{\ast}\right){K}_{m,t}^2+{\rho}_m^{\ast }{\overline{K}}_{m,t}^2 $$

Then the error between the mean of experiment ${\overline{E}}_{m,t}$ and the corresponding true expression value can be measured by means of the shrinkage error

8 $$ {\psi}_{m,t}=\sqrt{\frac{W_{m,t}^{\ast }}{R(t)}}. $$

Apparently, by introducing the parameter *R*(*t*) denoting the number of replicates for tissue *t*, different numbers of replicates are allowed for different experiments. If the number of replicates is the same for all experiments, then ${\psi}_{m,t}=\varepsilon {W}_{m,t}^{\ast }$, where *ε*=1/*R*(*t*).

Substituting the mean square error ${K}_{m,t}^2$ with the mean of the shrinkage error *ψ*_*m*, *t*_ in eq. (2), the shrinkage error- weighted average expression of poly(A) site *m* in experiment *t* is:

9 $$ {\overline{E}}_m^{SCCA}={\sum}_{t=1}^T\frac{{\overline{E}}_{m,t}}{\psi_{m,t}^2}/{\sum}_{t=1}^T\frac{1}{\psi_{m,t}^2}. $$

Therefore, the correlation coefficient of the *m*^th^ and *n*^th^ poly(A) site is [63]:

10 $$ {\lambda}_{mn}=\frac{\sum_{t=1}^T\frac{\left({\overline{E}}_{m,t}-{\overline{E}}_m^{SCCA}\right)}{\uppsi_{m,t}}\ \frac{\left({\overline{E}}_{n,t}-{\overline{E}}_n^{SCCA}\right)}{\uppsi_{n,t}}}{\sqrt{\sum_{t=1}^T{\left(\frac{\left({\overline{E}}_{m,t}-{\overline{E}}_m^{SCCA}\right)}{\psi_{m,t}}\right)}^2{\sum}_{t=1}^T{\left(\frac{\left({\overline{E}}_{n,t}-{\overline{E}}_n^{SCCA}\right)}{\psi_{n,t}}\right)}^2}} $$

Then correlation coefficient matrices of poly(A) sites within specific gene(s) are:

11 $$ \mathrm{corr}\left(P,Q\right)=\left[\begin{array}{c}{\sum}_{PP}\ {\sum}_{PQ}\ \\ {}\ {\sum}_{QP}\ {\sum}_{QQ}\ \end{array}\right]. $$

$$ {\sum}_{\mathrm{PP}}=\left[\begin{array}{ccc}{\lambda}_{11}& \cdots & {\lambda}_{1m}\\ {}\vdots & \ddots & \vdots \\ {}{\lambda}_{m1}& \cdots & {\lambda}_{mm}\end{array}\right],{\sum}_{QQ}=\left[\begin{array}{ccc}{\lambda}_{11}& \cdots & {\lambda}_{1n}\\ {}\vdots & \ddots & \vdots \\ {}{\lambda}_{n1}& \cdots & {\lambda}_{nn}\end{array}\right], $$

$$ {\sum}_{PQ}=\left[\begin{array}{ccc}{\lambda}_{11}& \cdots & {\lambda}_{1n}\\ {}\vdots & \ddots & \vdots \\ {}{\lambda}_{m1}& \cdots & {\lambda}_{mn}\end{array}\right],{\sum}_{QP}=\left[\begin{array}{ccc}{\lambda}_{11}& \cdots & {\lambda}_{1m}\\ {}\vdots & \ddots & \vdots \\ {}{\lambda}_{n1}& \cdots & {\lambda}_{nm}\end{array}\right], $$

The Lagrange multiplier method can be used to solve problem (1) [35, 64], then

12 $$ {\displaystyle \begin{array}{c}\left({\sum}_{QP}{\sum}_{PP}^{-1}{\sum}_{PQ}-{\xi}^2{\sum}_{QQ}\right){\boldsymbol{D}}_{\boldsymbol{Q}}=0\\ {}\left({\sum}_{PQ}{\sum}_{QQ}^{-1}{\sum}_{QP}-{\xi}^2{\sum}_{PP}\right){\boldsymbol{D}}_{\boldsymbol{P}}=0,\end{array}} $$

where *ξ*^2^ is the eigenvalue of matrix $P={\sum}_{PP}^{-1}{\sum}_{PQ}{\sum}_{QQ}^{-1}{\sum}_{QP}$. An alternative way to obtain the maximum value of *ξ* is to solve the matrix to get *k* positive eigenvalues

13 $$ {\xi}_1^2\ge {\xi}_2^2\ge \cdots \ge {\xi}_k^2, $$

where *k* = min {*m*, *n*}.

*ξ*_1_ is the first canonical correlation coefficient, which reflects the greatest degree of correlation. *ξ*_*k*_ is the *k*^th^ canonical correlation coefficient. Next we test the significance of each canonical correlation coefficient by using a hypothetical test based on the maximum likelihood criterion [65] to obtain statistically significant canonical correlation coefficients.

14 $$ {\displaystyle \begin{array}{c}{H}_0:{\xi}_1={\xi}_2=\cdots ={\xi}_k=0\\ {}{H}_1:{\xi}_1\ne 0\ \mathrm{or}\ {\xi}_2\ne 0\ \mathrm{or},\cdots, {\xi}_k\ne 0\ \mathrm{and}\ {\xi}_1\ge {\xi}_2\ge \cdots \ge {\xi}_k>0.\end{array}} $$

Given a sufficiently large *g*, the statistic of likelihood ratio for the *j*^th^ canonical correlation coefficient (jϵ[0, k]) is

15 $$ {\chi}_j^2=-\left[g-\frac{1}{2}\left(m+n\right)\right]{\sum}_{i=1}^k\log \left(1-{\xi}_i^2\right). $$

The canonical correlation coefficients follow the *χ*^2^-distribution with (*m* − *j*)(*n* − *j*) degrees of freedom.

Given a confidence interval (e.g., *α* = 0.05), if *j* = 0 and the null hypothesis is accepted, then *ξ*_1_ = 0 indicates that the two sets of variables are uncorrelated. If the null hypothesis is rejected, then it means that at least one of the canonical correlation coefficients is greater than 0, therefore the first pair of canonical variables is considered as significantly correlated. The hypothesis test is continued in the same way to verify whether the second canonical correlation coefficient is significant or not. This process is repeated until all non-zero canonical correlation coefficients are found.

For each pair of gene, at most *k* non-zero canonical correlation coefficients can be obtained. Although the first canonical correlation coefficient reflects the greatest degree of correlation between the two genes, solely using the first coefficient will neglect contributions of other canonical correlation coefficients. Here we used *p*-values of all non-zero coefficients from the hypothetical test to obtain the weight for each pair of gene that quantifies the degree of correlation [35].

16 $$ w=\frac{\sum_1^k{\xi}_kL\left(\log {P}_k\right)}{\sum_1^kL\left(\log {P}_k\right)} $$

where $$ L\left(\log P\right)=\left\{\begin{array}{c}0,P>0.05\\ {}-\log P,P\le 0.05\end{array}\right. $$. *P*_*k*_ is the p-value from the hypothetical test for the *k*^th^ correlation coefficient.

### Cluster analysis and network inference

Weights of all gene pairs obtained from PASCCA are first transformed to a similarity matrix, then the matrix is further used for clustering and network inference. In this study, we adopted the widely-used clustering method, hierarchical clustering, to cluster genes, which was implemented by the R function *hclust* with default parameters. WGCNA (v1.51) [39] was used to infer network modules (parameters: softPower = 6; mergeCutHeight = 0.05, minModuleSize = 30). Various metrics were employed to evaluate the clusters and modules obtained from different methods.

### Implementation of PASCCA

PASCCA is available as an R package, which is available for download via https://github.com/BMILAB/PASCCA. Computations in this study were carried out on a desktop computer with configuration “Intel(R) Core(TM) i5-4460T CPU @ 1.90GHz, and 8G RAM”. For practical application purpose for large scale data, we have leveraged the MPI (Message Passing Interface) framework to run PASCCA in parallel across many cores and nodes, which could drastically reduce the computing time. This package allows users to quantify associations between genes with/without repeated measurements using their own poly(A) site data and conduct downstream cluster analysis and network inference to explore important APA specific biological mechanism.

## Additional files

Additional file 1: Supplemental Figures. This file contains all the Supplemental Figures. (PPTX 206 kb)

Additional file 2: Hub genes for all gene modules obtained from PASCCA (XLSX 107 kb)

## Abbreviations

3’ UTR: 3′ untranslated region
ACC: Average clustering coefficient
AD: Average distance
ADM: Average distance between means
APA: Alternative polyadenylation
BHI: Biological homogeneity index
CCA: Canonical correlation analysis
CON: Connectivity
DUNN: Dunn index
EC: Eigenvector centrality
FPKM: Fragments per kilobase per million mapped fragments
GO: Gene ontology
HC: Hierarchical clustering
KEGG: Kyoto encyclopedia of genes and genomes; GSH: glutathione
MD: Module degree
mRNA: Messenger RNA
PASCCA: Cluster analysis of poly(A) site data using canonical correlation analysis
PCC: Pearson’s correlation coefficient
SCC: Shrinkage correlation coefficient
SD: Standard deviation
SDCC: Standard deviation weighted correlation coefficient
