# Supplementary material for: Cluster analysis of replicated alternative polyadenylation data using canonical correlation analysis
Source: BMC Genomics. 2019 Jan 22;20:75. doi: 10.1186/s12864-019-5433-7 (PMC6343338; doi:10.1186/s12864-019-5433-7)
Supplement: Supplementary file 1 — Supplemental Figures. This file contains all the Supplemental Figures. (PPTX 206 kb) [file 12864_2019_5433_MOESM1_ESM.pptx]

## Slide 1
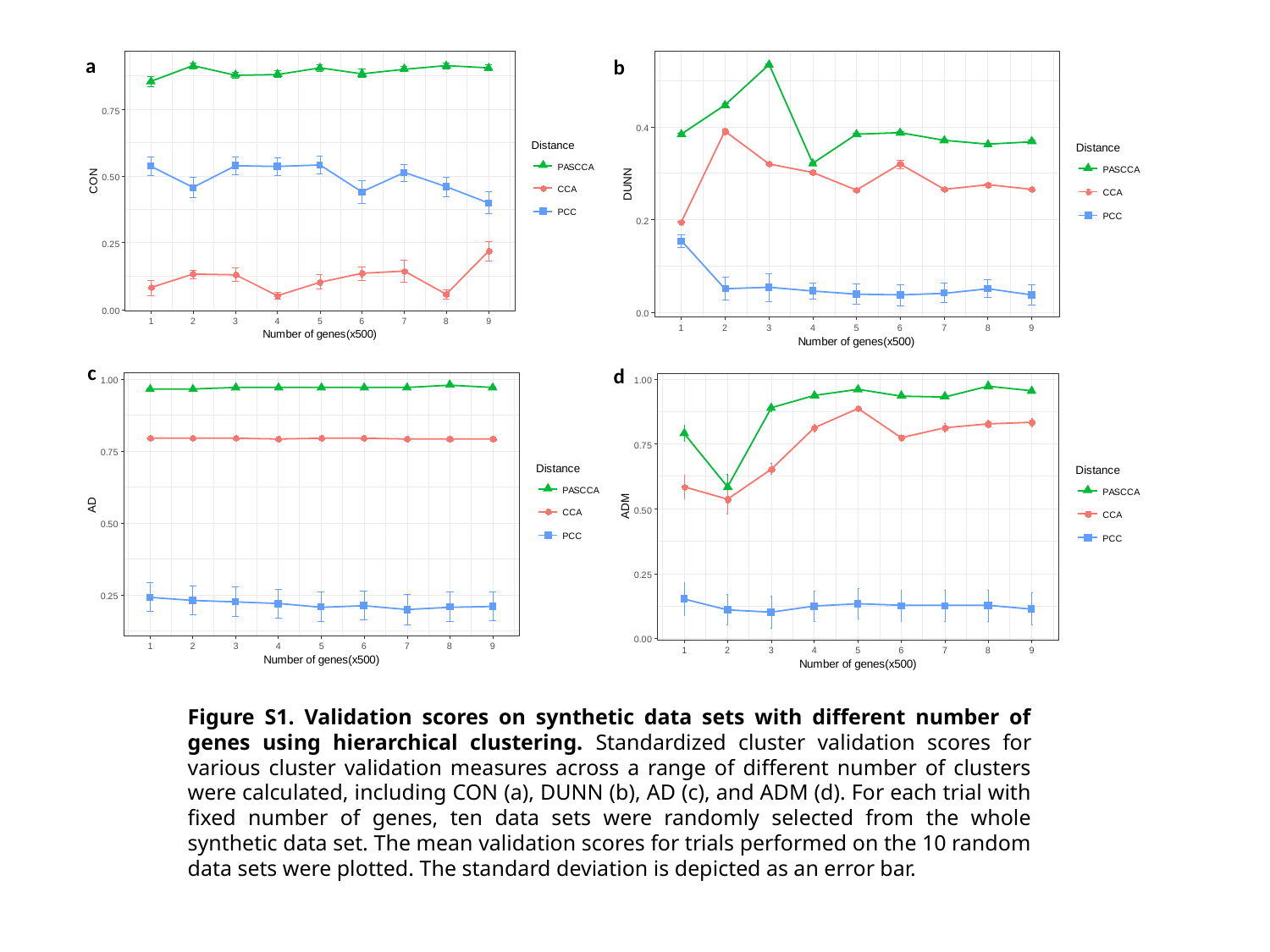

a
b
c
d
Figure S1. Validation scores on synthetic data sets with different number of genes using hierarchical clustering. Standardized cluster validation scores for various cluster validation measures across a range of different number of clusters were calculated, including CON (a), DUNN (b), AD (c), and ADM (d). For each trial with fixed number of genes, ten data sets were randomly selected from the whole synthetic data set. The mean validation scores for trials performed on the 10 random data sets were plotted. The standard deviation is depicted as an error bar.

## Slide 2
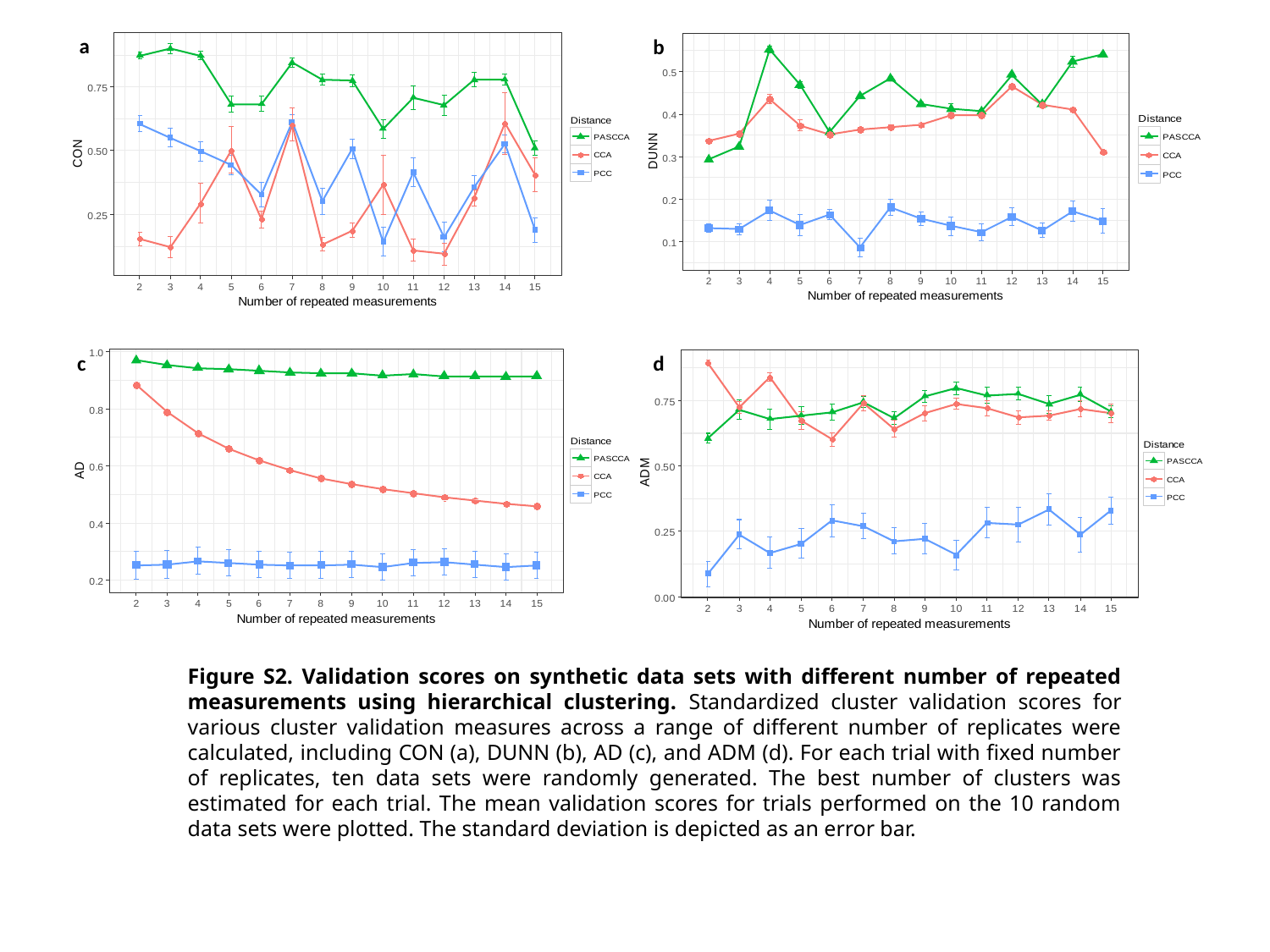

a
b
c
d
Figure S2. Validation scores on synthetic data sets with different number of repeated measurements using hierarchical clustering. Standardized cluster validation scores for various cluster validation measures across a range of different number of replicates were calculated, including CON (a), DUNN (b), AD (c), and ADM (d). For each trial with fixed number of replicates, ten data sets were randomly generated. The best number of clusters was estimated for each trial. The mean validation scores for trials performed on the 10 random data sets were plotted. The standard deviation is depicted as an error bar.

## Slide 3
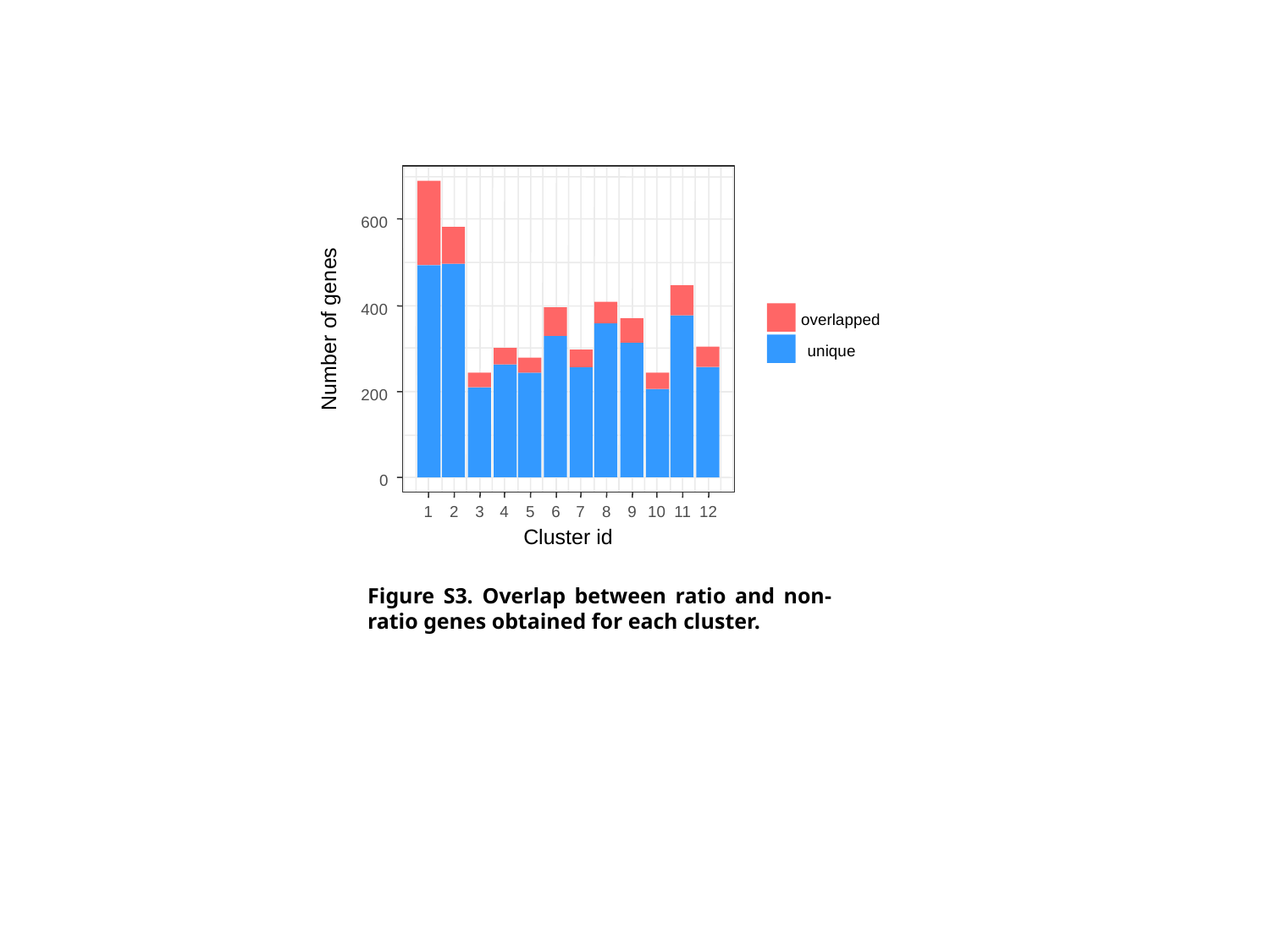

600
400
overlapped
Number of genes
unique
200
0
1
2
3
4
5
6
7
8
9
10
11
12
Cluster id
Figure S3. Overlap between ratio and non-ratio genes obtained for each cluster.

## Slide 4
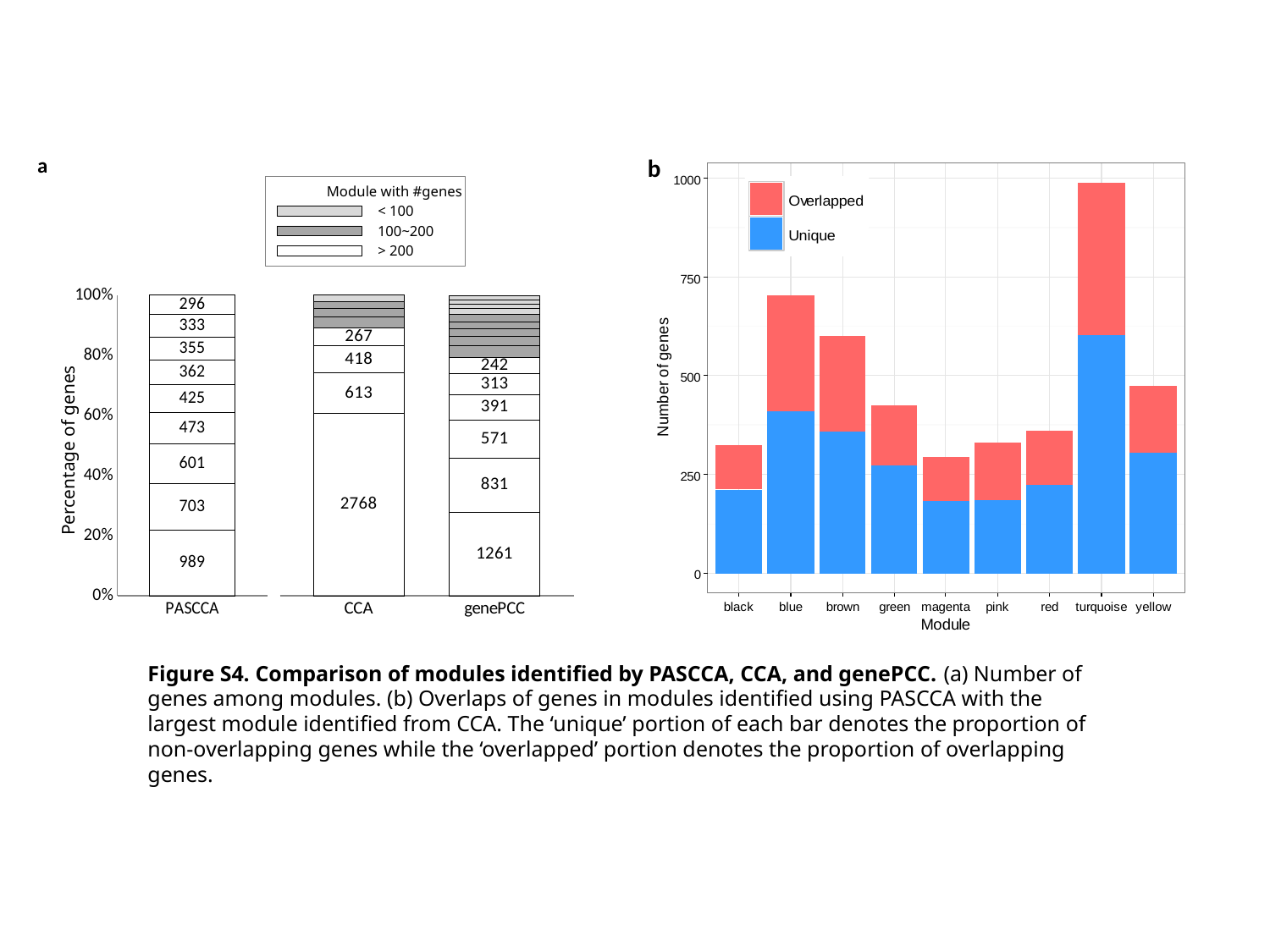

a
b
Module with #genes
< 100
100~200
> 200
### Chart
| Category | turquoise | blue | brown | yellow | green | red | black | pink | magenta |
|---|---|---|---|---|---|---|---|---|---|
| PASCCA | 989.0 | 703.0 | 601.0 | 473.0 | 425.0 | 362.0 | 355.0 | 333.0 | 296.0 |
### Chart
| Category | cyan | blue | brown | black | red | pink | greenyellow | salmon |
|---|---|---|---|---|---|---|---|---|
| CCA | 2768.0 | 613.0 | 418.0 | 267.0 | 164.0 | 136.0 | 102.0 | 96.0 |
### Chart
| Category | lightgreen | black | brown | green | midnightblue | grey60 | red | purple | greenyellow | tan | cyan | lightcyan | darkred | royalblue | darkgreen |
|---|---|---|---|---|---|---|---|---|---|---|---|---|---|---|---|
| genePCC | 1261.0 | 831.0 | 571.0 | 391.0 | 313.0 | 242.0 | 191.0 | 129.0 | 119.0 | 111.0 | 106.0 | 90.0 | 68.0 | 68.0 | 65.0 |Percentage of genes
Figure S4. Comparison of modules identified by PASCCA, CCA, and genePCC. (a) Number of genes among modules. (b) Overlaps of genes in modules identified using PASCCA with the largest module identified from CCA. The ‘unique’ portion of each bar denotes the proportion of non-overlapping genes while the ‘overlapped’ portion denotes the proportion of overlapping genes.

## Slide 5
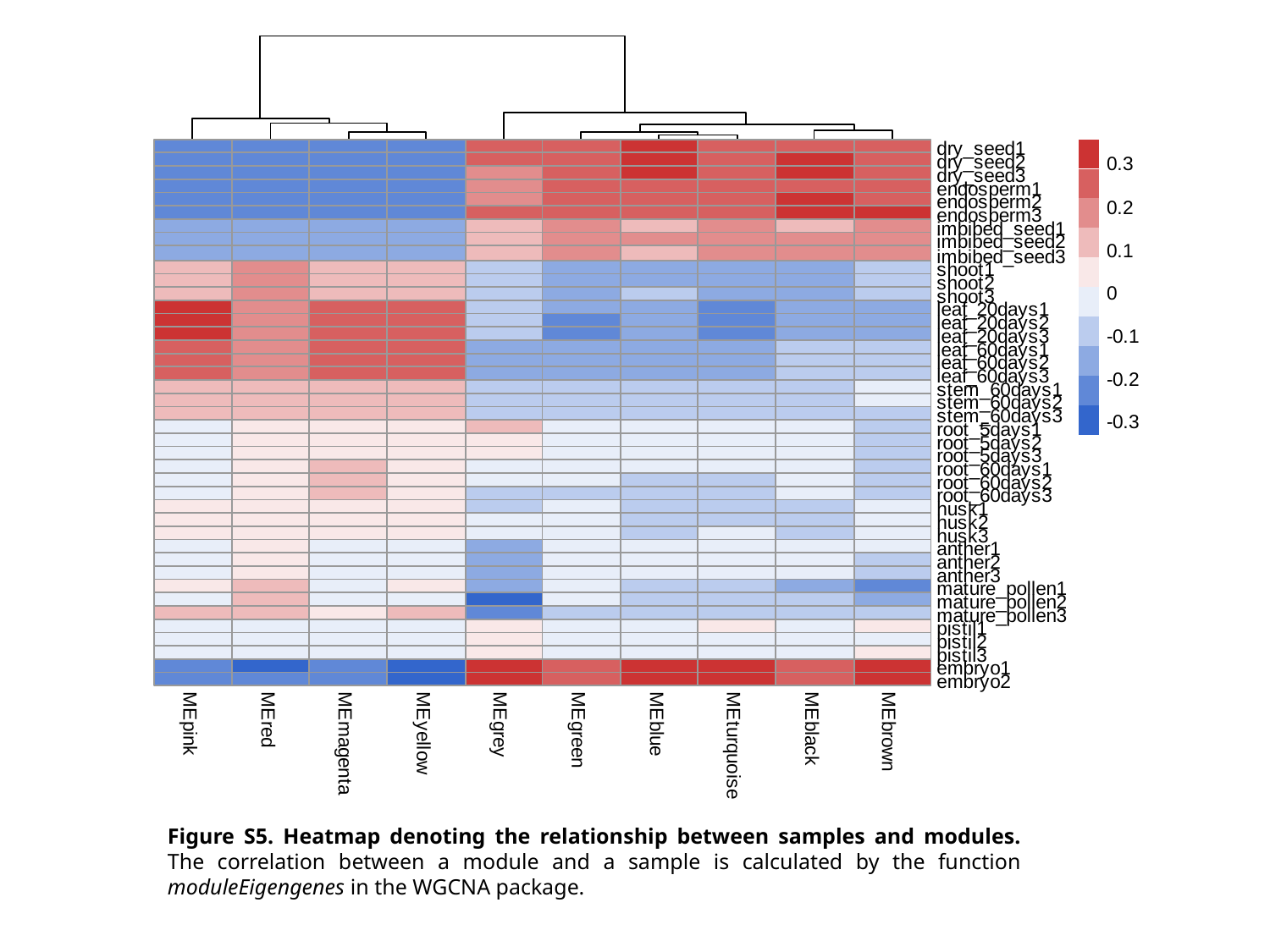

Figure S5. Heatmap denoting the relationship between samples and modules. The correlation between a module and a sample is calculated by the function moduleEigengenes in the WGCNA package.
